# The improvement of fibrinogen, Ang-1, VEGF, BDNF in post-operative patients with brain trauma through target task-oriented phase training

**DOI:** 10.5937/jomb0-45490

**Published:** 2024-06-15

**Authors:** Bo Liu, Huan Yu

**Affiliations:** 1 The First Affiliated Hospital of Harbin Medical University, Neurosurgery Department, Harbin, China; 2 The 242nd Hospital Harbin, Rehabilitation Medicine Department, Harbin, China

**Keywords:** target task-oriented phase training, post-operative brain trauma, Fbg, Ang-1, VEGF, BDNF, quality of life, ciljani zadati trening, postoperativna povreda mozga, Fbg, Ang-1, VEGF, BDNF, kvalitet života

## Abstract

**Background:**

It aims to explore the effect of target task-oriented phase training on fibrinogen (Fbg), angiopoietin (Ang-1), vascular endothelial growth factor (VEGF), serum brain-derived neurotrophic factor (BDNF), and quality of life in post-operative patients with brain trauma.

**Methods:**

142 patients with brain trauma who were operated on in neurosurgery of our hospital from March 2020 to March 2023 were chosen and separated into two groups by random number table. The control group (n=71) received routine post-operative training. The experimental group (n=71) received target task-oriented training based on the control group, and the serum cell levels of nursing for 3, 7, and 14 days were compared. Improvement of limb function and quality of life after 2, 4, and 6 weeks of nursing care is observed.

## Abstract (sr)

**Uvod:**

Cilj ovog istraživanja je bio da se ispita efekat ciljanih zadatih treninga u fazi rehabilitacije na nivoe fibrinogena (Fbg), angiopoetina-1 (Ang-1), vaskularnog endotelijalnog faktora rasta (VEGF), serumskog faktora neurotrofnog mozga (BDNF) i na kvalitet života kod pacijenata nakon operacije u vezi sa povredom mozga.

**Metode:**

Odabrano je 142 pacijenata sa povredom mozga koji su operisani na odeljenju neurohirurgije naše bolnice od marta 2020. do marta 2023. godine i koji su podeljeni u dve grupe slučajnim izborom. Kontrolna grupa (n=71) je imala rutinske postoperativne treninge, dok je eksperimentalna grupa (n=71) dobijala ciljane zadate treninge na osnovu kontrolne grupe, a nivoi faktora u serumu su praćeni tokom 3, 7 i 14 dana. Posmatrano je poboljšanje funkcije udova i kvaliteta života posle 2, 4 i 6 nedelja nege.

**Rezultati:**

Pre nege, upoređivanje nivoa faktora u serumu, rezultata za funkciju udova i rezultata za kvalitet života između dve grupe bilo je sa P>0,05. Nakon 3, 7 i 14 dana nege, Fbg u kontrolnoj grupi je bio viši nego u eksperimentalnoj grupi. Nivoi Ang-1, VEGF i BDNF u eksperimentalnoj grupi su bili viši nego u kontrolnoj grupi (P<0,05). Nakon 2, 4 i 6 nedelja nege, FMA rezultati za gornje i donje udove u kontrolnoj grupi su bili niži nego u eksperimentalnoj grupi, sa P<0,05. Rezultati na fiziološkom, psihološkom, socijalnom polju i na polju životnog okruženja kod kontrolne grupe su bili niži nego u eksperimentalnoj grupi, sa P<0,05.

**Zaključak:**

Primena ciljanih zadatih treninga u fazi rehabilitacije kod pacijenata sa povredom mozga nakon operacije može da pomogne u podizanju nivoa Fbg, Ang-1, VEGF i BDNF u serumu, da poboljša funkciju udova i unapredi kvalitet života.

## Introduction

Brain trauma is the injury of brain tissue, nerves, and blood vessels caused by external forces. According to a global public health survey, the annual incidence rate of brain trauma ranks highest among traumatic diseases, reaching up to 35% [1]. Domestic data surveys also indicate a significant incidence rate of 21%, posing a substantial threat to people’s health and lives.

Currently, clinical craniotomy is a crucial method for treating brain trauma. However, the complex nature of brain tissue results in longer recovery periods for post-operative tissues and nerves. Some patients experience varying degrees of neurological dysfunction after surgery, leading to alterations in serum levels, limb dysfunction, and a reduced quality of life [2]. Research has shown that effective neurological and limb function training can aid in restoring serum levels, promoting limb function recovery, and reducing disability rates in patients following brain trauma surgery [3].

Despite traditional nursing care for brain trauma ensuring a smooth treatment process, there is often insufficient emphasis on limb rehabilitation training, and the absence of standardized training plans hinders significant improvement in promoting limb function recovery [4]. Therefore, implementing effective, scientific, and targeted training and rehabilitation programs is paramount for post-operative patients with brain trauma.

Targeted Task-Oriented Phase Training is a nursing training approach developed for post-operative rehabilitation in brain trauma patients. It considers the unique characteristics of the patient’s recovery stages and formulates specific training objectives and measures for each recovery phase. This approach is pivotal in promoting overall functional recovery [5].

Naeeni Davarani et al. [6] conducted a study involving phased rehabilitation training in post-operative brain trauma patients, focusing on improving serum factor levels, enhancing limb function recovery, and increasing overall life abilities. This project selected brain trauma patients as the subjects and analyzed the training impact during the Targeted Task-Oriented Stage implementation. The following report outlines the findings of this study.

## Materials and methods

### Research materials

A total of 142 post-operative patients with brain trauma were included in the study. They were randomly assigned to either a control group (CG) or an experimental group (EG), with 71 patients in each group. Gender, age, cause of injury, Glasgow Outcome Scale (GOS) score, and educational background information were compared, and the results indicate that all p-values exceeded 0.05, as displayed in Table 1.

**Table 1:** Comparison of general information between two groups (x̄±s,%).

Group	n	Gender		Age <br>(years)	Etiology of injury (%)		
		Male	Female		Traffic accident	Falling injury	Tumble injury
CG	71	46	25	56.48±10.43	37	18	16
EG	71	42	29	56.81±10.07	35	22	14
*t*		0.478		0.192	0.589		
*p*		0.489		0.84	0.745		
Group	n	GOS Rating (%)			Educational background		
		Grade III	Grade	Grade	Primary and junior <br>high school	High school and technical <br>secondary school	College degree <br>or above
CG	71	26	31	14	2	32	37
EG	71	24	35	12	4	35	32
*t*		0.476			1.163		
*p*		0.788			0.559		

### Inclusion criteria and exclusion criteria

Inclusion criteria: (A) Brain trauma is confirmed by imaging CT examination and in line with the relevant diagnostic criteria of the Chinese neurosurgery expert consensus on the diagnosis and treatment of primary brain stem hemorrhage; (B) Surgical treatment is performed for the first time in our hospital; (C) The surgery is smooth, and the post-operative condition and vital signs are stable; (D) Patients with post-operative complications of limb dysfunction; (E) Cognitive function, language function, and mental state are normal and cooperation with research is required.

Exclusion criteria: (A) Combined limb joint and motor function injuries during trauma; (B) Post-operative complications such as infection, epilepsy, language function, and cognitive impairment; (C) Previous history of nervous system disease; (D) Severe osteoporosis, unable to cooperate with training implementation; (E) Merge malignant tumors of the body, such as lung cancer, liver cancer, gastric cancer, etc.

### Research methods

CG (Control Group): Routine nursing care is provided. The patient’s condition and vital signs are closely monitored and recorded after surgery. Medication is administered following medical advice. The patient must remain in bed and receive regular repositioning and back tapping to maintain a clean and dry bed environment. Respiratory secretions are promptly managed, and oxygen inhalation or phlegm suctioning is performed if necessary. As advised by medical professionals, once the patient’s condition improves, passive and active limb function training is initiated.

EG (Experimental Group): Targeted task-oriented phase training is implemented, divided into three stages:

Brain Edema Stage: Task objectives are established. The primary training tasks in this phase involve passive training and maintaining the body’s functional position. Passive training is guided by the charge nurse, who instructs the patient’s family members to perform joint exercises, including flexion, extension, internal rotation, external rotation, and abduction on the patient’s upper and lower limbs. The training starts from proximal joints and progresses to distal joints. Large joints are trained before small joints, and unaffected limbs are trained before the affected side. Each joint is exercised 20 times once a day. Functional position training includes positioning the patient in a side-lying position with specific limb and hand placement instructions. The position is changed at specified intervals during the day and night.

Stage of Stable Condition: In this stage, the primary objective is active limb training. Patients are encouraged to perform exercises to lift and move their limbs. Activities include bed rolling, sitting-up exercises, and joint flexion and extension exercises. Major joints, including fingers, toes, hips, knees, and elbows, are trained with 20 repetitions twice daily.

Disease Recovery Period: This stage aims to achieve independent daily life activities. Patients are assisted in creating a daily off-bed training plan, recording methods, frequency, times, and precautions. Patients and their families are instructed to engage in bedside and corridor walking according to the plan. Close monitoring of the patient’s face, heart rate, and breathing is essential during training, with immediate cessation in case of abnormalities. Patient tolerance is regularly evaluated, and gradual assistance is provided for daily activities such as dressing, eating, face-washing, and bathroom use. Nursing care is administered once a day, lasting 15-30 minutes each time and continuing until patient discharge.

### Observation indicators and evaluation

Serum Index Levels: The attending physician collaborates with the laboratory to assess fibrinogen (Fbg), angiopoietin-1 (Ang-1), vascular endothelial growth factor (VEGF), and brain-derived neurotrophic factor (BDNF) before commencing nursing care and on the 3rd, 7th, and 14th days of care. A 3 mL venous blood sample is collected and centrifuged at 3000 revolutions per minute for 10 minutes. Fibrinogen (Fbg) levels are determined using turbidimetry, while Ang-1 and VEGF levels are measured through enzyme-linked immunosorbent assays. BDNF levels are assessed utilizing the chemiluminescence method.

Limb Function: The attending physician assists the charge nurse in conducting assessments using the Fugl-Meyer (FM) motor function assessment scale at 2, 4, and 6 weeks into the nursing care. This scale comprises five dimensions: motor function, pain, body sensation, joint range of motion, and balance function. With a total of 113 items, the scale employs a 0-3 level scoring method, resulting in a maximum score of 226 points. This study focuses on the motor function dimension, consisting of two parts: 33 items for upper limb FM and 17 items for lower limb FM, all scored on a 0–2-point scale. The total scores range from 0-66 points for upper limb function and 0-34 points for lower limb function, with higher scores indicating improved limb function recovery.

Quality of Life: Charge nurses evaluate the quality of life using the World Health Organization Quality of Life Brief Scale (WHO QOLBREF), developed by the World Health Organization, at 2, 4, and 6 weeks during nursing care. The scale comprises 29 items, covering individual aspects such as health status, quality of life, appetite, self-assessment, and family relationships. The remaining 24 items are categorized into four dimensions: physiological, environmental, psychological, and social fields. Scores are calculated based on the scale’s scoring principles.

### Data statistics processing

Two individuals input the data into the database and process it using the statistical software package SPSS 25.0. We utilize 2 tests to analyze count data between groups, describing the results using the number of cases (n) and percentages (%). The independent sample t-test is employed for between-group comparisons, and the results are presented as mean ± standard deviation (M±SD). A significance level of P < 0.05 indicates a statistically significant difference in the data comparison.

## Results

### Comparison of general information between two groups

The comparison in the cause of injury, GOS score grading, and others in Table 1 was with *P*>0.05.

### Comparison of serum indicator levels between two groups before and after 3, 7, and 14 days of nursing care

**Figure 1:**
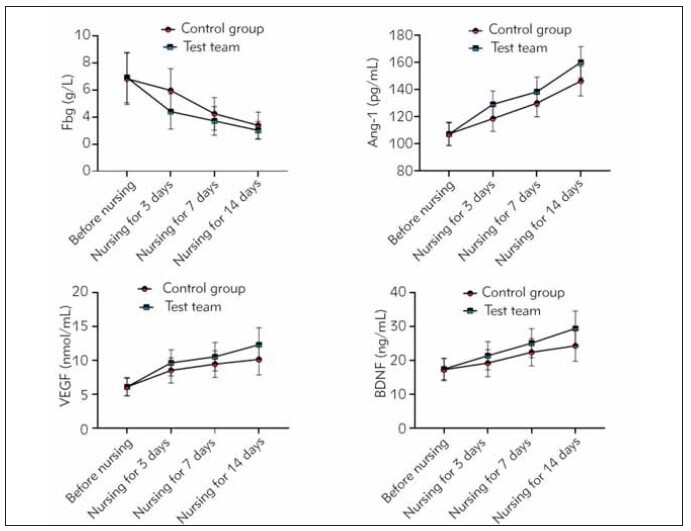
Comparison of various serum factor indicators between the two groups before and after 3, 7, and 14 days of nursing care.

Before nursing, the comparison in serum levels was with *P*>0.05. After 3 days, 7 days, and 14 days of nursing care, Fbg in both groups decreased, and the EG was lower than the CG. The levels of Ang-1, VEGF, and BDNF in both groups increased, and the EG was higher than the CG, with *P*<0.05, as expressed in Table 2. Figure 1

**Table 2:** Levels of various serum indicators before and after 3, 7, and 14 days of care in two groups (x̄±s). Note*: Compared to before nursing in this group, *P*<0.05; #: Compared to this group, *P*<0.05 after 3 days of nursing care; BDNF: Brain-derived neurotrophic factor; &: Compared to this group, after 7 days of nursing care, *P*<0.05; Fbg: Fibrinogen; Ang-1: Angiopoietin; VEGF: Vascular endothelial growth factor.

Group	n	Fbg, g/L				Ang-1, pg/mL			
		Before nursing	Nursing 3d	Nursing 7d	Nursing 14d	Before nursing	Nursing 3d	Nursing 7d	Nursing 14d
CG	71	6.84±1.91	5.98±1.61*	4.25±1.21*#	3.39±0.98*#&	107.45±8.67	118.68±9.33*	130.15±10.12*#&	146.34±10.99*#&
EG	71	6.93±1.86	4.41±1.30*	3.72±1.06*#	3.02±0.67*#&	107.29±8.25	129.27±9.78*	138.46±10.71*#&	160.18±11.42*#&
*t*		0.284	5.936	2.776	2.626	0.113	6.602	4.752	7.358
*p*		0.777	0.000	0.006	0.010	0.911	0.000	0.000	0.000
Group	n	VEGF, nmol/mL				BDNF, ng/mL			
		Before nursing	Nursing 3d	Nursing 7d	Nursing 14d	Before nursing	Nursing 3d	Nursing 7d	Nursing 14d
CG	71	6.14±1.37	8.56±1.86*	9.48±1.97*#	10.18±2.30*#&	17.35±3.26	19.27±3.99*	22.46±4.01*#	24.38±4.57*#&
EG	71	6.13±1.28	9.67±1.92*	10.57±2.10*#	12.34±2.51*#&	17.51±3.18	21.43±4.15*	25.16±4.26*#	29.48±5.13*#&
*t*		0.045	3.499	3.190	5.346	0.296	3.161	3.889	6.254
*p*		0.964	0.001	0.002	0.000	0.768	0.002	0.000	0.000

### Comparison of limb FM scale scores between two groups before nursing, 2, 4, and 6 weeks of nursing care

**Figure 2:**
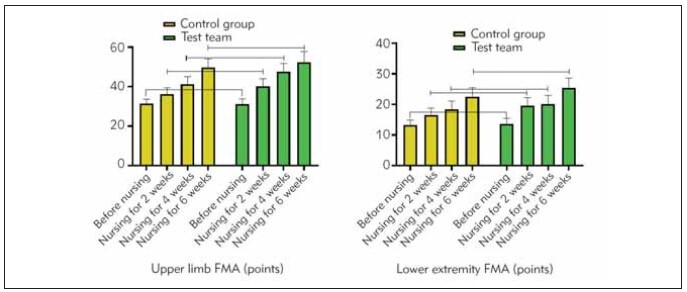
Before nursing, 2, 4, and 6 weeks of nursing for two groups of limb FM scale scores.

Before nursing, the comparison in overall limb function scores had *P*>0.05. After 2, 4, and 6 weeks of nursing care, the comparison in the upper limb and lower limb FMA scores had P<0.05, as displayed in Table 3. Figure 2

**Table 3:** Two groups of limb FM scale scores before nursing, 2, 4 and 6 weeks of nursing (x̄±s, points).

Group	n	Upper limbFMA				Lower limbsFMA			
		Before <br>nursing	Nursing for <br>2 weeks	Nursing for <br>4 weeks	Nursing for <br>6 weeks	Before <br>nursing	Nursing for <br>2 weeks	Nursing for <br>4 weeks	Nursing for <br>6 weeks
CG	71	31.48±2.19	36.24±3.27*	41.26±3.81*#	49.67±4.37*#&	13.27±1.67	16.48±2.31*	18.49±2.63*#	22.48±3.02*#&
EG	71	31.16±2.72	40.27±3.76*	47.57±4.17*#	52.34±5.46*#&	13.64±1.82	19.68±2.55*	20.16±2.84*#	25.48±3.15*#&
*t*		1.062	6.815	9.413	3.217	1.262	7.837	3.635	5.793
*p*		0.290	0.000	0.000	0.002	0.209	0.000	0.000	0.000

### Comparison of various scores on the WHOQOL-BREF scale between two groups before nursing, 2, 4, and 6 weeks after nursing

**Figure 3:**
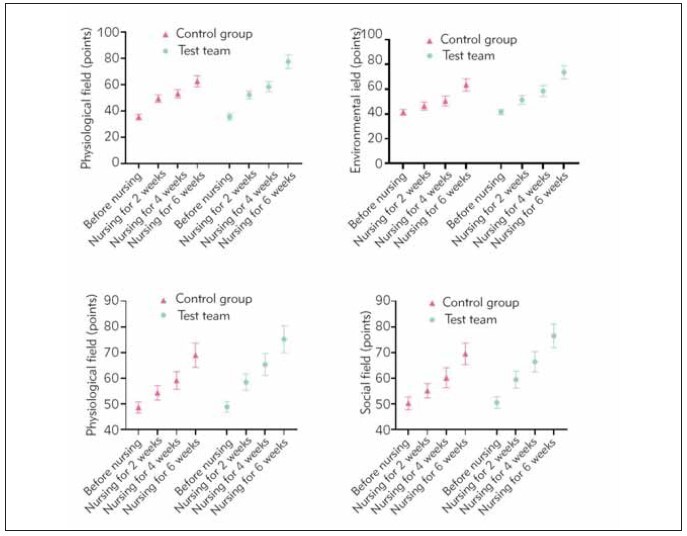
Scores of the WHOQOL-BREF scale before nursing, 2, 4, and 6 weeks after nursing in two groups.

Before the commencement of nursing care, the comparison of WHOQOL-BREF scale scores indicated no significant difference with P > 0.05. However, following 2, 4, and 6 weeks of nursing care, scores in both groups increased in the physiological, environmental, psychological, and social domains. The Experimental Group exhibited higher scores than the Control Group with P < 0.05, as indicated in Table 4. Figure 3

**Table 4:** Each score of the WHOQOL-BREF scale before nursing, 2, 4, and 6 weeks after nursing in two groups (x̄±s, points). Note*: Compared to before nursing in this group, *P*<0.05; #: Compared to this group, *P*<0.05 after 3 days of nursing care; &: Compared to this group, after 7 days of nursing care, *P*<0.05; WHO QOL BREF: Quality of life scale developed by the World Health Organization.

Group	n	Physiological field				Environmental field			
		Before <br>nursing	Nursing for <br>2 weeks	Nursing for <br>4 weeks	Nursing for <br>6 weeks	Before <br>nursing	Nursing for <br>2 weeks	Nursing for <br>4 weeks	Nursing for <br>6 weeks
CG	71	35.48±2.16	49.48±2.77*	53.18±3.15*#	62.67±4.16*#&	41.36±2.26	46.38±3.18*	50.49±3.98*#	63.45±4.87*#&
EG	71	35.61±2.48	52.37±2.96*	58.55±3.82*#	77.67±5.16*#&	41.58±2.02	51.27±3.57*	58.48±4.27*#	73.67±5.26*#&
*t*		0.333	6.007	9.139	19.069	0.612	8.618	11.534	12.013
*p*		0.740	0.000	0.000	0.000	0.542	0.000	0.000	0.000
Group	n	Psychological field				social field			
		Before <br>nursing	Nursing for <br>2 weeks	Nursing for <br>4 weeks	Nursing for <br>6 weeks	Before <br>nursing	Nursing for <br>2 weeks	Nursing for <br>4 weeks	Nursing for <br>6 weeks
CG	71	48.67±2.16	54.36±2.78*	59.16±3.45*#	68.98±4.68*#&	50.34±2.46	55.16±2.82*	60.19±3.85*#	69.54±4.16*#&
EG	71	48.93±2.04	58.49±3.15*	65.37±4.26*#	75.15±5.24*#&	50.57±2.19	59.49±3.24*	66.48±3.96*#	76.48±4.65*#&
*t*		0.737	8.283	9.454	7.400	0.588	8.494	9.596	9.373
*p*		0.462	0.000	0.000	0.000	0.557	0.000	0.000	0.000

## Discussion

### The significance of implementing target task-oriented phase training after brain trauma surgery

Traffic accidents and violent injuries are important causes of brain trauma. Craniotomy is often required for the dangerous disease. Although surgery can alleviate the state of illness and improve the neurological function of brain tissue, it has a poor effect on disease rehabilitation [7]. The study of Ho et al. [8] indicated that there is a high incidence of neurological deficits and motor dysfunction after brain trauma surgery, which affects patients’ normal limb function and reduces their ability to live. However, the brain central nervous tissue has remodeling and regeneration physiological characteristics. If it is not given timely and comprehensive rehabilitation training at all stages of tissue and nerve recovery after surgery, the surgical effect will be reduced, leading to the aggravation of neurological deficit and limb dysfunction. Naeeni Davarani et al. [6] studied that implementing phased rehabilitation training in the early stage after brain trauma surgery is crucial for promoting limb function recovery, reducing disease disability rates, and improving quality of life. It is necessary to strengthen training interventions for neurological function and limb motor function in patients with brain trauma after surgery.

Target task-oriented phase training is a training mode based on the training tasks and targets of each phase of disease rehabilitation, which is in line with the process of post-traumatic brain injury rehabilitation in each stage and strictly follows the principle of sequential training [5]. The post-operative rehabilitation effect of patients with brain trauma is reflected in the improvement of their condition through surgical treatment, gradually assisting in the recovery of neurological and limb functions through effective and scientific training modes. Training regulates the excitatory state of nerves by stimulating the neurons of the body’s motor pathway at the optimal time for recovery at each stage, thereby promoting normal motor output and the recovery of neurological and limb motor functions [9]. Target task-oriented phase training has important significance in post-operative patients with brain trauma.

### The effect of target task-oriented training on post-operative Fbg, Ang-1, VEGF, and BDNF in patients with brain trauma

Fibrinogen (Fbg) is a protein crucial for coagulation and primarily synthesized by the liver, serving as a precursor to fibrin. Additionally, it is identified as coagulation factor I, the most abundant plasma coagulation factor. Fbg plays a vital role in various physiological processes, including interactions with plasmin, thrombin, and coagulation factor XIIIa as a substrate [10]. When considered a separate variable, fibrinogen serves as a prognostic indicator for the overall condition of patients with traumatic brain injuries. Managing fibrinogen levels within the range of 2.5 to 3 grams per liter could potentially enhance the clinical outcomes of individuals suffering from TBI [11]. Our study showed increased fibrinogen levels following task-oriented training in brain injury patients, which could contribute to the beneficial effects of this training in rehabilitating these patients.

Ang-1 is a type of vascular growth factor that can serve as a ligand for the receptor of arginine kinase, playing a role in the vascular system and closely related to vascular stability and development. Previous animal model studies by Zheng et al. [12] showed that enhancing Ang-1 and Tie-2 expression through exercise improves the recovery of brain function. Similarly, our study demonstrated that targeted task-oriented training significantly increases Ang-1 levels.

VEGF is also a kind of vascular endothelial growth factor that can increase vascular permeability, change the properties of the extracellular matrix, promote the migration of endothelial cells in blood vessels, and thus rebuild new vascular structures [13] [14]. BDNF is a protein value that can nourish nerves and is widely expressed in the nervous system. Elevated levels of BDNF can promote neuronal recovery, enhance adaptability in the body, and play an important role in promoting neural recovery [15] [16]. The findings of this study indicated that the serum Fbg levels of patients in the EG who received targeted task-oriented training for 3, 7, and 14 days were lower than those in the CG who received routine care. The VEGF and BDNF levels in the EG were higher than those in the CG, which is consistent with the results of Chaturvedi et al. [17]. One primary reason for these differences is that target task-oriented training is more systematic and focused than conventional training, enhancing the safety and efficacy of the training process. By implementing training programs tailored to the specific needs of patients at various stages of brain trauma (such as brain edema, stable, and recovery stages), we directly stimulate limb muscles, indirectly enhance muscle blood circulation, facilitate venous and lymphatic vessel compression through muscle contractions and joint movement, and effectively promote the return of lymphatic and venous fluids, thereby improving local blood circulation and reducing stasis.

Furthermore, effective training can enhance local nerve excitability, bolster synaptic plasticity, encourage synaptic conduction function recovery, and aid in recovering damaged brain tissue. This stimulation results in the secretion of various cytokines, including Ang-1, VEGF, BDNF, and others, ultimately enhancing multiple key indicators.

### The impact of target task-oriented training on limb function and quality of life after brain trauma surgery

Limb function and quality of life are critical assessment criteria for evaluating the post-operative rehabilitation outcomes in patients with brain trauma. Improvements in limb function and enhanced quality of life indicate significant progress in patient prognosis and rehabilitation outcomes.

The results of this study reveal that, compared to the Control Group (CG), patients in the Experimental Group (EG) who received targeted taskoriented phase training experienced an increase in quality-of-life scores after 2, 4, and 6 weeks of nursing care. Findings from studies such as Alsubiheen et al. [18] have shown that implementing phase balance training in patients with brain injuries resulting from chronic stroke not only enhances limb function recovery but also leads to an improved quality of life. Our study aligns with these research findings.

This positive impact can be attributed to several factors. Firstly, passive training and functional position maintenance for brain edema patients with brain trauma effectively prevent joint stiffness and deformity resulting from prolonged bed rest and immobilization. This approach helps maintain muscle metabolism and lays the foundation for stable and rehabilitation training. Patients are guided to transition from passive to active training during the stable period. This transition involves repeated stimulation of motor nerve pathways, forming new neural pathways, reconstructing damaged brain nervous tissue, and improving nerve function, ultimately enhancing limb motor function [19] [20].

Physical medicine and physiotherapy play a pivotal role in rehabilitating and recovering after a traumatic brain injury (TBI) [3]. Following a TBI, individuals often experience a wide range of physical impairments, such as muscle weakness, balance issues, and coordination difficulties, which can significantly impact their overall quality of life. Physiotherapy and physical medicine interventions are essential in helping TBI patients regain their physical function and independence [21]. Through carefully tailored exercise programs and therapeutic techniques, physiotherapists work to improve strength, mobility, and motor skills, helping patients relearn basic activities of daily living and regain their confidence. Additionally, physiotherapy can address pain management and assist in preventing secondary complications that may arise due to immobility. Ultimately, the importance of physical medicine and physiotherapy in the aftermath of a traumatic brain injury cannot be overstated, as they are vital components in the comprehensive, holistic approach to TBI rehabilitation, enabling individuals to maximize their recovery and regain their independence [3] [12] [21] [22].

Furthermore, during the rehabilitation phase, activities such as bed exercises and daily training assist patients in reinforcing the correct movement patterns in their brains. This enhances the excitability, sensitivity, and reactivity of neural activities, promotes the recovery of various bodily functions, increases daily living abilities, and improves overall quality of life.

In summary, implementing targeted task-oriented phase training in patients with brain trauma holds significant clinical value. This approach contributes to restoring brain tissue neural function, enhancing Fbg, Ang-1, VEGF, BDNF levels, and improving limb function and overall quality of life.

## Dodatak

### Conflict of interest statement

All the authors declare that they have no conflict of interest in this work.
